# Data on MyoD reduction by autophagy in C2C12 cells

**DOI:** 10.1016/j.dib.2017.06.034

**Published:** 2017-06-24

**Authors:** Yeong-Min Yoo, Yung Chul Park

**Affiliations:** a Department of Forest Environment Protection, College of Forest and Environmental Science, Kangwon National University, Chuncheon, Gangwon-do 24341, Republic of Korea; b Institute of Forest Science, College of Forest and Environmental Science, Kangwon National University, Chuncheon, Gangwon-do 24341, Republic of Korea

**Keywords:** MyoD, Degradation, Autophagy, C2C12 cells

## Abstract

Autophagy is a highly regulated physiologic mechanism in which cells maintain homeostasis by degrading excessive or unnecessary proteins and damaged or aged organelles through the lysosomal machinery (Yorimitsu and Klionsky, 2005) [1]. MyoD is basic helix-loop-helix (bHLH) transcription factors that regulate myoblast proliferation and myogenic differentiation. MyoD is expressed in adult skeletal muscle (Megeney et al., 1996) [2] and adult fibers (Brack et al., 2005) [3]. MyoD is mainly degraded by the ubiquitin-proteasome system (Floyd et al., 2001) [4] and partly by autophagy (Kim et al., 2012) [5]. Data showed that autophagy decreased MyoD protein in C2C12 cells by Western blotting analysis.

## **Specifications Table**

**Table:** 

Subject area	*Biology*
More specific subject area	*Cell biology*
Type of data	*Figures*
How data was acquired	*Western blotting analysis, real-time PCR*
Data format	*Analyzed*
Experimental factors	*Autophagy in C2C12 cells was induced by treatment of high fetal bovine serum (FBS).*
Experimental features	*MyoD degradation by autophagy showed Western blotting under high concentrations of FBS.*
Data source location	*Chuncheon, Gangwon-do, Republic of Korea*
Data accessibility	*All data are available with this article*

## **Value of the data**

• This data could give a base for the detection of MyoD protein in both muscle cells and C2C12 cells by Western blotting analysis.
• The data will be useful for investigating that nutrition oversupply including high concentration of FBS may increase autophagy in both muscle cells and C2C12 cells.
• The data allow us to promote that regulation of MyoD protein may suppress myoblast proliferation and myogenic differentiation.

## 1 Data

**Fig. 1:**
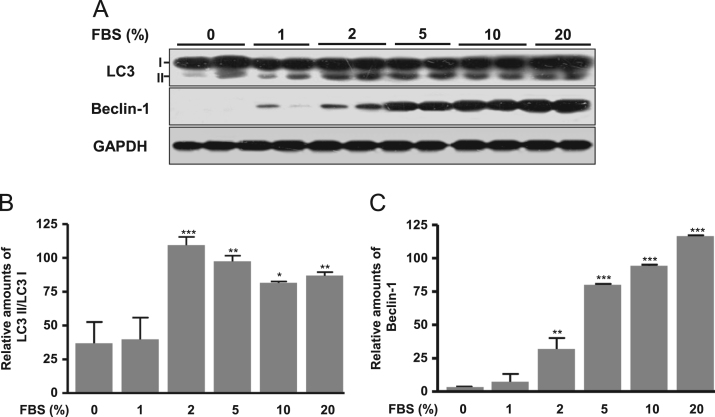
Expression of LC3 (A, B) and Beclin 1 proteins (A, C) in C2C12 cells. Data represent mean±SD of three experiments. *p<0.05, **p<0.01, ***p<0.001 vs. 0% FBS.

**Fig. 2:**
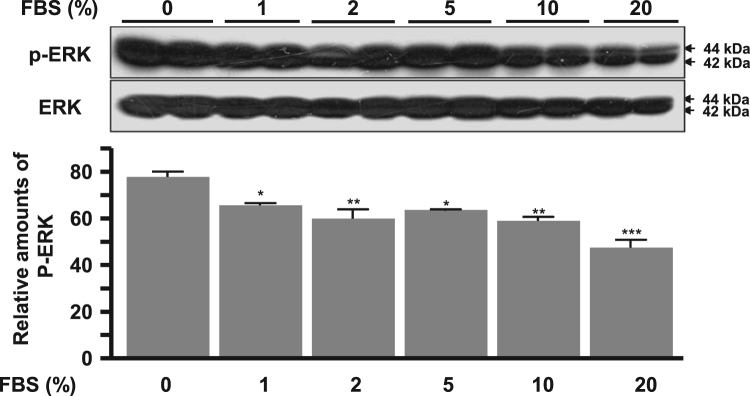
Expression of p-ERK in C2C12 cells. Data represent mean±SD of three experiments. *p<0.05, **p<0.01, ***p<0.001 vs. 0% FBS.

**Fig. 3:**
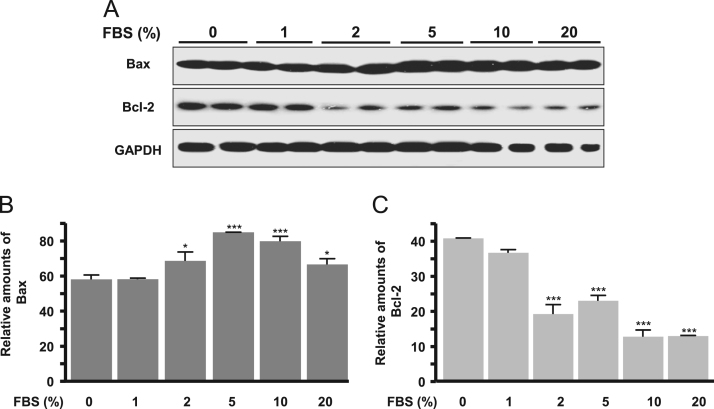
Expression of Bax (A, B) and Bcl-2 proteins (A, C) in C2C12 cells. Data represent mean±SD of three experiments. *p<0.05, ***p<0.001 vs. 0% FBS.

**Fig. 4:**
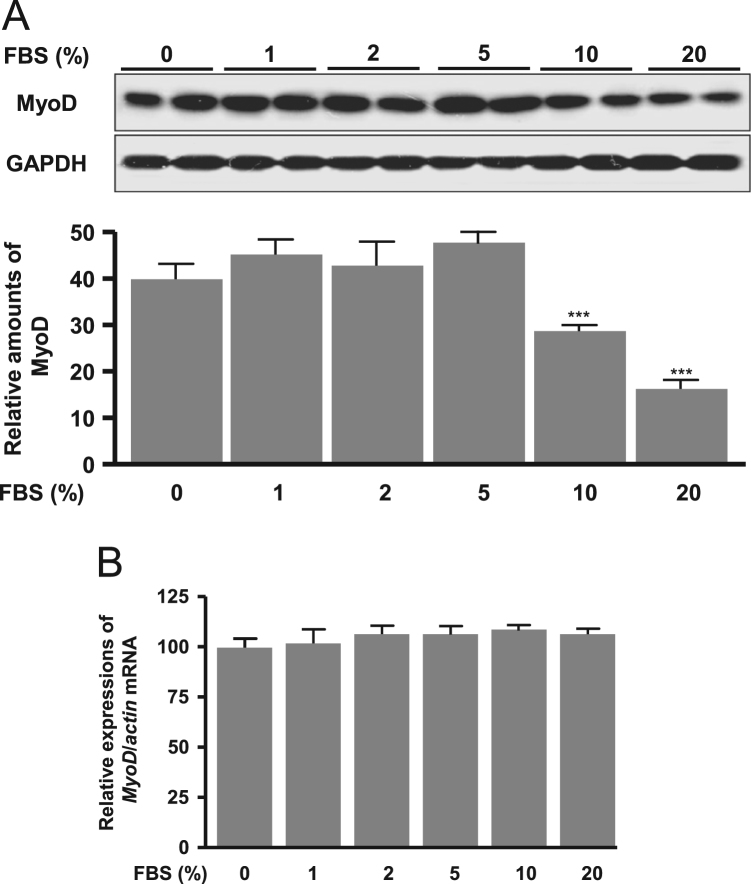
Expression of MyoD protein (A) and mRNA (B) in C2C12 cells. Data represent mean±SD of three experiments. ***p<0.001 vs. 0% FBS.

**Fig. 5:**
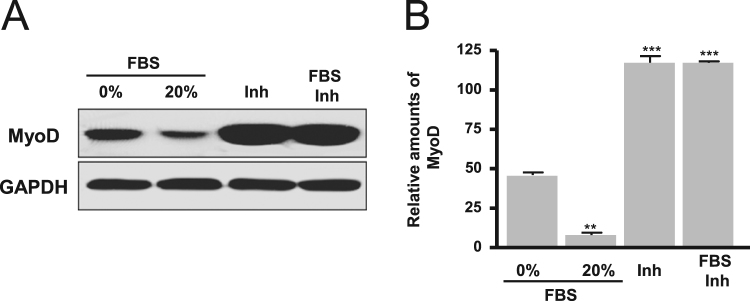
MyoD expression with autophagy inhibitor bafilomycin A1 in C2C12 cells. Data represent mean±SD of three experiments. **p<0.01, ***p<0.001 vs. 0% FBS. Inh, autophagy inhibitor bafilomycin A1.

The autophagy was increased by treatment of dose-dependent FBS (1–20%) and a subsequent autophagy markers, LC3II and Beclin-1 proteins significantly increased (Fig. 1). Cell proliferation signal phospho-ERK significantly decreased according to dose-dependent FBS (Fig. 2). Proapoptotic molecule Bax protein expression was increased in more than 5% FBS treatments compared to the absence of FBS and antiapoptotic molecule Bcl-2 protein expression was reduced in more than 2% FBS treatments (Fig. 3). Under the same conditions, cytosolic MyoD protein was significantly decreased in 10 and 20% FBS condition (Fig. 4A), but MyoD mRNA did not change (Fig. 4B). C2C12 cells were treated with autophagy inhibitor bafilomycin A1, and then completely blocked degradation of MyoD (Fig. 5). Together, these results suggest that high FBS-induced autophagy results in degradation of MyoD protein in C2C12 myoblast cells.

## 2 Experimental design, materials and methods

### 2.1 Cell culture

We performed as described previously [5]. C2C12 myoblast cells were cultured in Dulbecco׳s modified Eagle׳s medium (DMEM, GibcoBRL, Gaithersburg, MD, USA) with 5% fetal bovine serum (FBS, GibcoBRL) at 37 °C with 5% CO_2_. C2C12 cells were incubated in DMEM containing 1–20% FBS and/or with 0.1 μM autophagy inhibitor bafilomycin A1 (Calbiochem, San Diego, MO, USA) for 24 h.

### 2.2 Western blot analysis

We performed as described previously [6]. Cells with 80–90% confluence was prepared using buffer (150 mM NaCl, 1% NP-40, 50 mM Tris–HCl, pH 7.4, 0.1 mM phenylmethylsulfonyl fluoride, 5 µg/mL aprotinin, 5 µg/mL pepstatin A, 1 µg/mL chymostatin, 5 mM Na_3_VO_4_, and 5 mM NaF), incubated for 30 min at 4 °C, and centrifuged at 13,000×*g* for 20 min at 4 °C. Proteins (40 µg) were separated by 12% sodium dodecyl sulfate-polyacrylamide gel electrophoresis. Antibodies were LC3, Beclin 1, p-ERK, ERK, Bax, Bcl-2, MyoD, and GAPDH from Santa Cruz Biotechnology (Santa Cruz, CA, USA). Protein bands measured using ImageJ software (version 1.37; Wayne Rasband, NIH, Bethesda, MD, USA) and normalized to GAPDH.

### 2.3 RNA extraction and quantitative real-time PCR

We performed as described previously [7]. Total RNA was prepared using Trizol reagent (Invitrogen, Carlsbad, CA, USA). cDNA template (2 μL) was analyzed in triplicate by addition of 10 μL 2× SYBR® Premix Ex Taq^TM^ (TaKaRa Bio. Inc., Otsu, Shiga, Japan) using a 7300 Real-time PCR System (Applied Biosystems, Foster, CA, USA): denaturation at 95 °C for 5 min, 40 cycles of denaturation at 95 °C for 30 s, annealing at 60 °C for 30 s, and extension at 72 °C for 45 s. The primers were *MyoD*, 5′-AGTGAATGAGGCCTTCGAGA-3′ (sense) and 5′-GCATCTGAGTCGCCACTGTA-3′ (antisense); *β-actin*, 5′-AGCCATGTACGTAGCCATCC-3′ (sense) and 5′-TTTGATGTCACGCACGATTT-3′ (antisense). Fluorescence intensity threshold was taken as the threshold cycle in the exponential phase of PCR amplification. Relative expression was calculated using the equation R=2^-[ΔCT sample-ΔCT control]^.

### 2.4 Statistical analysis

Significant differences were determined by ANOVA using the Prism Graph Pad v4.0 (Graph Pad Software Inc., San Diego, CA, USA). *P* values < 0.05 were considered statistically significant.

## Funding sources

This research was supported by 2014 Research Grant from Kangwon National University (No. 120141458) and Basic Science Research Program through the National Research Foundation of Korea (NRF) funded by the Ministry of Education (2016R1A6A3A11934650).
